# Acknowledgment to the Reviewers of *Methods and Protocols* in 2022

**DOI:** 10.3390/mps6010009

**Published:** 2023-01-16

**Authors:** Methods and Protocols Editorial Office

**Affiliations:** MDPI AG, St. Alban-Anlage 66, 4052 Basel, Switzerland

High-quality academic publishing is built on rigorous peer review. *Methods and Protocols* was able to uphold its high standards for published papers due to the outstanding efforts of our reviewers. Thanks to the efforts of our reviewers in 2022, the median time to first decision was 24 days and the median time to publication was 50 days. Regardless of whether the articles they examined were ultimately published, the editors would like to express their appreciation and thank the following reviewers for the time and dedication that they have shown *Methods and Protocols*:
Agata StanekLijo Cherian OzhathilAhmed Eisa ElhagLinda RykkjeAinhoa AgorretaLinda S. EdelmanAlberto YuferaLisa M. JenkinsAlejandro Santos-LozanoLouis Jun Ye OngAlessandro NotaLucas Dos Santos DiasAlexander LangLuxin SunAlexandra BogomazovaMagda Ecaterina AntohéAlexia KagiavaMałgorzata GniewoszÁlvaro MaciasMałgorzata PihutAmin DerouicheMallesh KurakulaAmy NoserMara Pavel-DinuAna Maria IordacheMaria Isabel VeigaAnca RoşeanuMarin SenilaAndre WirriesMarina P. IsaevaAndreas NerlichMarta SzachniukAndreja Trojner-BregarMaryam BoshtamAndrew J. SmithMasafumi NodaAnna BarcelóMateusz WaliczekAnna ImpallariaMatteo Bruno LodiAnnarita StringaroMaximiliano Luis CacicedoAntonio Ignacio Martín-GarcíaMehdi KoushkiArnaud PallottaMeng SunAshkan RashadMichael MillerAzhwar RaghunathMiroslav BarancikBeata Morak-MłodawskaMiroslav JůzlBeatriz Buentello-VolanteMonika KosmalaBertrand SimonMorgan BroggiBilbao Gaskon IbarretxeMuhammad IqhrammullahBozena KaminskaMun-Ho RyuBrenda SealsNadine WiesmannCamille EsneauNavdar SeverCarla FabroNicolai CraciunChang-Ye HuiNoemi GiannettaCharles Bou-NaderOllie Yiru YuChi-Wei ChenPantelis Varvaki RadosChris McConvillePaolo MeneguzzoChristine Seel RitchiePaul HooykaasChun-Gu ChengPaul S. AmieuxClaudia ColantonioPere Lavega-BurguésCristian RotariuPeter N. LipkeCristina GrippaudoPeter PikoDa SunPetra Lindholm-LehtoDafna SussmanPhilip HublitzDavid E. LevyPierluigi OnaliDebadrita PariaPierre TennstedtDeepika AwasthiPierre-Louis StengerDorin GheorghePing YipDoris RušićPolonowita AjithEdna NavaPorto Chiara LoEdyta PasickaPramod K. KalambateElena A. DomblidesRachel Spronken-SmithElena McBeathRafael Coutinho Mello MachadoEleonora MolestiRanjit ShresthaElisa BorsaniRanjith KumavathElisa ScalcoRine ReubenElisabeth DarjRoberto CodellaEmese GalRodney P. JonesEmilio ParisiniRonald Allan PanganibanEvert NjomenRuiying ZhaoFengjie SunSalvatore ScolavinoFeofilovs MaksimsSamuel Ken-En GanFerman KonukmanSandrine Bittencourt BergerFernando Capela E. SilvaSebastian BöttgerFlavia FiorilloSergio ChicumbeFlorence Awino OyiekeSergiy VelychkoGabrijela DumbovicSeth FrietzeGaurav KwatraShannon RoseGaurav V. SarodeShenqi WangGema FrühbeckShubhendu PaleiGeorge FragoulisSilvia CarvalhoGeorgios S. MarkopoulosSoichiro MurataGilberto De NucciSoňa LegartováGiulia FranzoniSonal ManoharGongchen SunSounak SahuGuillaume FalgayracSridhar MuthusamiGustavo Moreno-MartínStefan Holland-CunzHamdoon MohammedStéphane BlouinHassan ZafarStoyan PetkovHeinz-Dietrich WuttkeSubhas KhajanchiHiroki KatoSusanna SpinsanteHiromi SakaiSvetlana VorobyovaHumberto PrietoTakahito NakajimaInnokentii E. VishnyakovTao HuangIoannis IliopoulosTao YaoIrina B. KaradjovaTejal PatelIrina-Iuliana CostacheTeresa AlmeidaIssan ZhangTeruyoshi AmagaiJames SolomonTessa R. EnglundJasmine W. TayThomas LindnerJayasimha Rayalu DaddamTomas BogielJiri BarekTuhin RoychowdhuryJitender K. Kiran KumarVerena SchultzJonathan PerreaultVeronica GiuntiniJuan González-FernándezVictoria HandfordKalina HaasVictoria PastorKaruna DixitWei YangKatarina VukojevićWoongsik JangKatie TrottaXenia PenateKaushik GhoseXijun PiaoKeiko MiyoshiYanawath SantaladchaiyakitKonstantinos ChronisYaoguang LiuKristina PennistonYaoyao PengKuang-Ming KuoYongjin XuKumar Chandan SrivastavaYoselin Benitez-AlfonsoLamprini MalletzidouYuichiro NishidaLaura Antonio-ZancajoZhiming LinLeena P. BharathZongjie WangLeopoldo González-Brusi


